# Effect of a needs-based model of care on the characteristics of healthcare services in England: the i-THRIVE National Implementation Programme

**DOI:** 10.1017/S2045796025000101

**Published:** 2025-03-26

**Authors:** R Sippy, L Efstathopoulou, E Simes, M Davis, S Howell, B Morris, O Owrid, N Stoll, P Fonagy, A Moore

**Affiliations:** 1 Department of Psychiatry, University of Cambridge, Cambridge, UK; 2 Anna Freud, London, UK; 3 Department of Department of Clinical, Educational and Health Psychology, University College London, London, UK

**Keywords:** adolescents, epidemiology, health service research, mental health, psychiatric services

## Abstract

**Aims:**

Developing integrated mental health services focused on the needs of children and young people is a key policy goal in England. The THRIVE Framework and its implementation programme, i-THRIVE, are widely used in England. This study examines experiences of staff using i-THRIVE, estimates its effectiveness, and assesses how local system working relationships influence programme success.

**Methods:**

This evaluation uses a quasi-experimental design (10 implementation and 10 comparison sites.) Measurements included staff surveys and assessment of ‘THRIVE-like’ features of each site. Additional site-level characteristics were collected from health system reports. The effect of i-THRIVE was evaluated using a four-group propensity-score-weighted difference-in-differences model; the moderating effect of system working relationships was evaluated with a difference-in-difference-in-differences model.

**Results:**

Implementation site staff were more likely to report using THRIVE and more knowledgeable of THRIVE principles than comparison site staff. The mean improvement of fidelity scores among i-THRIVE sites was 16.7, and 8.8 among comparison sites; the weighted model did not find a statistically significant difference. However, results show that strong working relationships in the local system significantly enhance the effectiveness of i-THRIVE. Sites with highly effective working relationships showed a notable improvement in ‘THRIVE-like’ features, with an average increase of 16.41 points (95% confidence interval: 1.69–31.13, P-value: 0.031) over comparison sites. Sites with ineffective working relationships did not benefit from i-THRIVE (−2.76, 95% confidence interval: − 18.25–12.73, P-value: 0.708).

**Conclusions:**

The findings underscore the importance of working relationship effectiveness in the successful adoption and implementation of multi-agency health policies like i-THRIVE.

## Background

Mental healthcare has numerous well-supported models. Systems are challenged to provide appropriate services efficiently, necessitating reform of current care models (Hodgins *et al.*, 2024). The continuation of inadequate care is mainly due to difficulties in applying changes (Grol and Grimshaw, 2003; Shortell *et al.*, 1993), as systemic transformation is complex (Best *et al.*, 2012). In England, the transformative “Future in Mind” report (Department of Health, 2015) suggested deviating from tiered services to a more responsive model for the specific mental health needs of local young populations. It called for service providers and funders to overhaul children and young people’s mental health (CYPMH) services into comprehensive systems, offering services from prevention to risk management. The “Future in Mind” report focuses on reforming a system characterised by disunity, inefficiency, and limited access. Since 2016, Child and Adolescent Mental Health Services (CAMHS) within England’s National Health Service (NHS) have seen significant changes, advancing more comprehensive approaches to care (Rocks *et al.*, 2020).

The THRIVE Framework guides system reformation, summarising the needs of children and young people (CYP) into five groups: Getting Advice, Getting Risk Support, Getting Help, Getting More Help, and Thriving (Wolpert *et al.*, 2019). Support is provided to these CYP groups using a set of guiding principles of care, that encompass characteristics of support at three levels: macro, meso, and micro. Macro-level characteristics include interagency cooperation, meaning a 'THRIVE-like' CAMHS system would involve supporting bodies such as educational and social services in its policy-making and service delivery. Meso-level characteristics include a needs-based perspective focusing on CYP and support services. A site adhering to meso-level THRIVE principles would be expected to have a network of community providers. Micro-levelcharacteristics include interactions between CYP, their families, and healthcare professionals; we would expect shared decision-making, with everyone understanding the child’s needs and interventions (Moore *et al.*, 2023).

The National i-THRIVE Programme (NIP) assists CAMHS sites in adopting THRIVE principles, which prioritise patient needs and cohesive service provision through collaborative care networks (Moore *et al.*, 2023). Over 65% of CYP live in areas where i-THRIVE has been adopted (i-THRIVE Team 2023). i-THRIVE was created following implementation science guidelines to facilitate adoption of THRIVE principles by CAMHS (Moore *et al.*, 2016). The NIP study protocol has been published (Moore *et al.*, 2023). Briefly, i-THRIVE translates the complex aspects of a ‘THRIVE-like’ system into practical structures and tools for CAMHS transformation. The NIP guides CAMHS staff and leaders in developing local models based on THRIVE principles and creating detailed plans for implementation over four phases, using six components (Moore *et al.*, 2023). The implementation strategies in the NIP are drawn from the Quality Implementation Framework (Meyers *et al.*, 2012) and the Normalisation Processing Theory (May, 2006). The embodiment of THRIVE principles depends not only on efforts by CAMHS but also on broader community involvement (Wolpert *et al.*, 2019), requiring effective working relationships among local systems. The implementation strategies used in the NIP include use of evaluative and iterative strategies, providing interactive assistance, adaptation and tailoring to context, development of stakeholder interrelationships, training and education of stakeholders, engagement of consumers, utilisation of financial strategies, and changes to infrastructure (Waltz *et al.*, 2015).

Evaluation of implementation strategies is needed to understand the implementation’s impacts and determine if it should be adopted for use real-world. The evaluation could include several outcome metrics, such as acceptability, feasibility, fidelity or cost (Smith and Hasan, 2020). Fidelity is a particularly important metric, as it helps to determine if observed effects can be attributed to the intervention of interest; poor implementation may explain cases with no observed effects (Sanders *et al.*, 2022). In this work, we evaluate the impact of the NIP implementation model on fidelity to the THRIVE principles of care; a healthcare service unit that embodies the THRIVE principles of care would exhibit high fidelity to THRIVE at all levels.

Although in use since 2016, the NIP as an implementation model has not been assessed. We focus on the average effect of NIP on the THRIVE fidelity of sites. For this estimate, we need to know the outcome at each site if NIP had not been implemented (the potential outcome or unobserved counterfactual) (Stuart, 2010). The difference-in-differences (DiD) approach compares outcome changes between implementation and control sites before and after the intervention. A key assumption for DiD is that the average outcomes for both groups would have similar trends over time (Stuart *et al.*, 2014), which may not always be plausible. Participation in NIP is voluntary, meaning selection bias is possible. The four-group propensity score-weighted DiD method overcomes these issues by adjusting to ensures comparability among the groups and reduces bias in estimating the desired effect: particularly useful when group composition changes during the study as when health practitioners move between Clinical Commissioning Groups (CCGs).

In this study, we evaluate staff alignment with THRIVE principles and assess the effect of the NIP on THRIVE fidelity. We hypothesize that i-THRIVE had a positive effect on THRIVE fidelity. Additionally, we examine how the effectiveness of local system working relationships moderates the impact of the NIP on THRIVE fidelity.

## Methods

### Study setting and design

The study protocol is detailed elsewhere (Moore *et al.*, 2023). We selected 20 CAMHS sites in England: 10 sites using i-THRIVE since 2016 (NIP/implementation sites), and 10 using different transformation approaches (comparison sites). Details on NIP delivery are in Supplemental Material S1.

The NIP sites were Bexley, Cambridgeshire and Peterborough, Camden, Hertfordshire, Luton, Manchester, Stockport, Tower Hamlets, Waltham Forest and Warrington. The comparison sites included Bradford, Ipswich and East Suffolk, Lewisham, Norfolk, Northampton, Portsmouth, Southampton, Stoke-on-Trent, Sunderland and South Worcestershire. Sites are pseudonymised as Site A–T. Site details are in Supplementary Material S2. i-THRIVE started in April 2016; data from April 2015 to March 2016 were used as the pre-implementation period. Data from April 2018 to March 2019 were used as the post-implementation period. Surveys were used to examine staff alignment with and understanding of THRIVE principles, and to assess site transformation/implementation. To assess whether sites embraced THRIVE principles, fidelity scores were assigned. To understand whether the NIP impacted site adoption of THRIVE principles, fidelity scores from pre- and post-implementation were compared between implementation and comparison sites, while adjusting for site-level characteristics (auxiliary data).

### Measurements and data

#### Surveys

Two surveys were designed using the RE-AIM Adoption Framework (Glasgow *et al.*, 2019), a programme evaluation guide. Both surveys were primarily quantitative, with several open-ended questions (see Supplementary Materials S3–S5). The staff survey assessed staff awareness of THRIVE and use of THRIVE principles. Another survey was conducted among programme managers of i-THRIVE or comparison sites, gathering information about site transformation activities and received support (Supplementary Material S2).

#### Principal outcome: fidelity

Our primary focus is the degree to which sites follow the THRIVE principles of care, which encompass macro-, meso- and micro-level features and were assessed in sites using the i-THRIVE Assessment Tool (Moore *et al.*, 2023). Evaluators scored sites for overall fidelity (300 possible) and level-specific scores (macro: 84 possible, meso: 104 possible, micro: 112 possible), before and after the intervention. Higher scores indicate better THRIVE adherence. Additional details are in Supplementary Materials S2.

### Statistical analysis

We analysed the staff survey results using the chi-squared test and Cramer’s V as a measure of association between i-THRIVE/comparison sites and staff responses. We used a log-link binomial generalised linear model for site-specific results from ‘yes’/‘no’ responses, comparing the ‘yes’ probability for each site to the average among comparison sites.

The implementation lead survey responses are in Supplementary Material S1.

To assess inter-rater reliability for the fidelity scores, we used Krippendorf’s alpha (Supplemental Material S2).

Voluntary participation in health policy implementations like the NIP can lead to selection bias, as participating sites may differ from non-participating sites. To correct for this, we applied propensity score weighting (Rosenbaum and Rubin, 1985) to equalise the distribution of characteristics between the implementation and comparison sites. We used a four-group weighting method (pre-implementation i-THRIVE, post-implementation i-THRIVE, pre-implementation comparison, and post-implementation comparison) to align characteristics across all groups with those of the pre-implementation i-THRIVE sites (Stuart *et al.*, 2014). To calculate propensity score weights, it is important to identify site characteristics that might cause selection bias or have a confounding effect (Stuart *et al.*, 2014). Sources for these characteristics are in Supplementary Material S2.

Propensity scores were calculated using a multinomial model with five site characteristics (population density, annual funding, IMD rank, the number of CCGs per site, and transformation compliance); balance was checked using the standardised difference in means (Stuart, 2010). The impact of NIP on fidelity was estimated using maximum-likelihood repeated-measures linear regression with an auto-regressive correlation structure, weighted with calculated propensity scores. To account for remaining characteristic imbalances, we included population density, IMD, and transformation compliance in the model. These results represent the four-group-weighted DiD effect estimate.

To assess the reliability of our fidelity results, we conducted sensitivity analyses. We employed alternative methods to estimate the NIP effect, including the standard (unweighted) DiD and alternative model specifications. We examined the impact of non-compliant control sites by excluding these sites from analysis (Supplementary Material S6).

To examine variations in the effect of i-THRIVE, we investigated a possible effect moderator, specifically the quality of local system working relationships (NHS England, 2021) on i-THRIVE implementation (Supplementary Material S8).

## Results

### Surveys

The staff survey had 689 responses across 19 sites (no responses from Luton). Detailed results are in Supplementary Material S7. Although THRIVE Framework was widely known, more implementation respondents recognised it (83.9% vs. 70.5%, *P* < 0.0001). A higher proportion of implementation respondents reported using THRIVE principles in their daily practice (58.5% vs. 49.0%, *P* = 0.03), and scored perfectly on a test of THRIVE principles (34.1% vs. 22.9%, *P* = 0.001).

The transformation leads survey included eight managers from seven implementation sites and eight managers from seven comparison sites. Notably, managers from four comparison sites (Sites E, J, K and S) reported using THRIVE as their service transformation model. This prompted an examination of staff survey results concerning THRIVE implementation at comparison sites.

The site-level analysis of survey results can be found in Supplementary Material S7. Among comparison sites respondents, J and K reported a higher likelihood of implementing THRIVE. The odds of respondents reporting site implementation of THRIVE were 4.43 in J (95% CI: 2.32–8.47) compared to other comparison sites (76.5% vs. 59.5%), and 4.43 in K (95% CI: 1.33–14.80) compared to other comparison sites (76.5% vs. 59.5%). Among respondents at comparison sites, there was no difference in personal use of THRIVE principles compared to other comparison sites. Regarding knowledge of the THRIVE Framework, respondents from J had a higher probability of achieving a perfect score on the quiz: the odds of scoring perfectly were 3.73 (95% CI: 2.25–6.19), compared to other comparison sites (37.7% vs. 22.9%).

### THRIVE fidelity

**Figure 1.**
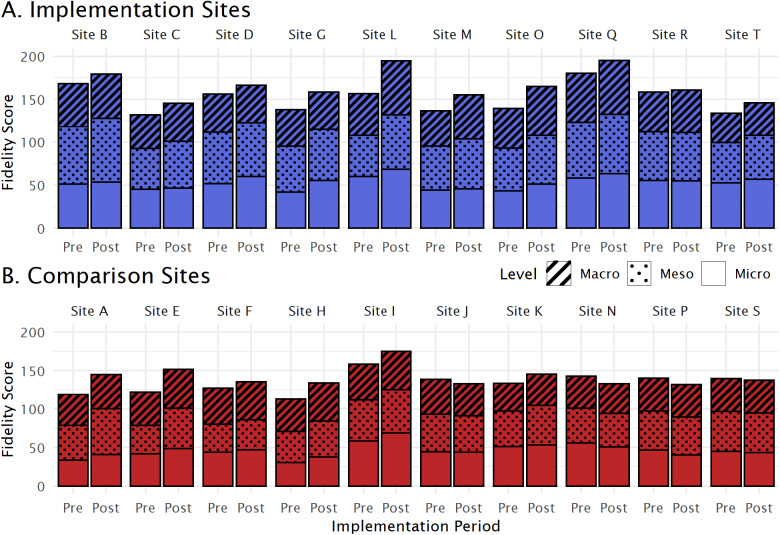
Fidelity scores by site. Total fidelity scores during the pre- and post-implementation periods are represented by bar height, with patterned overlay to indicate component levels (macro, meso, micro). Implementation sites are in panel a (blue) while comparison sites are in panel b (red).

**Figure 2.**
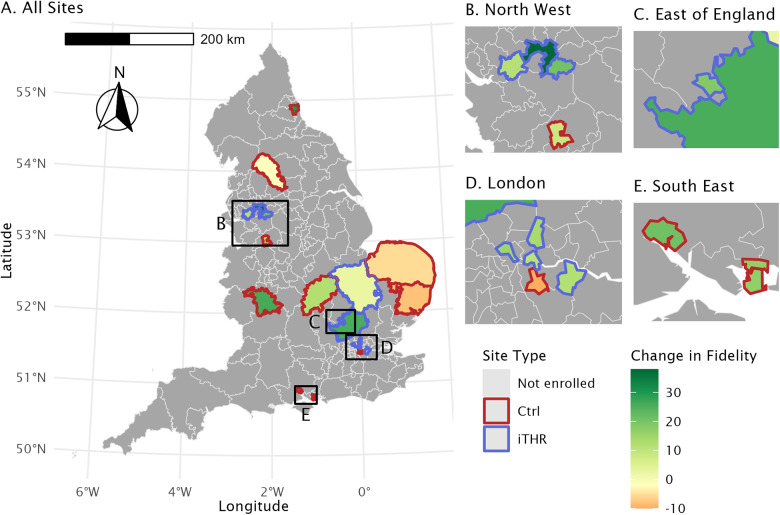
Change in fidelity scores over study period. The difference in total fidelity scores during the pre- and post-implementation periods are represented by colour, with an increased score in green and a decreased score in red, on a background map of clinical commissioning groups in (A). study sites have a bold outline (comparison sites in red, implementation sites in blue). inset maps for north west/midlands, London, and south east are in (B–D), respectively.

Inter-rater reliability for fidelity scores are in Supplementary Material S7. Before implementation, i-THRIVE sites had an average fidelity score of 149.0 (range: 132.0–180.2) and comparison sites averaged 133.4 (range: 113.0–158.2). Following implementation, i-THRIVE sites had an average score of 166.6 (range: 145.5–195.0), while comparison sites averaged 142.2 (range: 132.0–175.0). The mean difference between pre- and post-implementation among i-THRIVE sites was 16.7; among comparison sites the mean difference was 8.8. Two sites had incomplete fidelity score information: the macro-level components for Site T during the post-implementation period and the meso-level components for Site F during the pre-implementation period (scores were assigned as outlined in the ‘Methods’ section). Detailed fidelity scores by level and site are illustrated in Fig. 1; a map of the changes in scores by site is presented in Fig. 2.

Site characteristics, adjusted using four-group propensity-score weighting, are in Table S6, Supplementary Material S7. In the weighted standardised differences analysis, we identified some remaining imbalances (population density for the pre-implementation control group, IMD rank for both control groups, and transformation compliance for both control groups). These covariates were included in our effect estimate model, ensuring a more accurate assessment of NIP impact.

The NIP effect estimates are presented in Table 1. The overall fidelity scores were moderately influenced by the NIP. i-THRIVE sites showed an average improvement of 7.05 points (95% CI: − 4.47–18.57). The most notable improvements were at the macro-level, where i-THRIVE sites increased by an average of 2.92 points (95% CI: − 1.09–6.92), followed by the meso-level with an average increase of 2.76 points (95% CI: − 1.98–7.51), and the micro-level with an average increase of 1.39 points (95% CI: − 3.94–6.72). None of these improvements were statistically significant.

**Table 1.** Estimates of association between the national i-THRIVE programme and THRIVE fidelity

Outcome	Estimated score change	95% CI	*P*-value
Overall fidelity	7.05	−4.47 to 18.57	0.212
Macro-level fidelity	2.92	−1.09 to 6.92	0.141
Meso-level fidelity	2.76	−1.98 to 7.51	0.234
Micro-level fidelity	1.39	−3.94 to 6.72	0.588
CI = confidence interval.			

When comparing the four-group weighted DiD with the standard DiD analyses (Supplementary Material S6), we found comparable effects on overall and macro-level fidelity. There were shifts in the impacts on meso- and micro-level fidelity. This suggests that lower-level fidelity was more sensitive to the disparities between i-THRIVE and comparison groups, which were corrected through the four-group propensity-score weighting approach. Alternative modelling approaches produced results similar to our analysis. The exclusion of non-compliant comparison sites (i.e. Site J; see Supplementary Material S6) did not alter the results.

Among the study sites, five implementation sites and five comparison sites had highly effective working relationships among their local systems (Supplementary Material S8). We observed moderation by working relationship effectiveness on the impact of the NIP for overall and macro-level fidelity. i-THRIVE was found to be more effective at sites with highly effective working relationships. The detailed results are in Table 2. i-THRIVE sites with highly effective working relationships showed increased fidelity scores compared to comparison sites with highly effective working relationships. The most significant impact was on overall fidelity scores (16.41, 95% CI: 1.69–31.13), followed by macro-level scores (6.95, 95% CI: 2.15–11.75). The moderating influence of highly effective working relationships on meso-level and micro-level fidelity was modest (5.52, 95% CI: − 0.66–11.71 and 3.95 points, 95% CI: − 3.24–11.15, respectively). Notably, there was no discernible impact of the NIP on sites with ineffective working relationships across any fidelity level.

**Table 2.** Effect modification by working relationship quality on the impact of the national i-thrive programme

Outcome	Working relationship quality	Estimated score change	95% CI	*P*-value
Overall fidelity	Highly effective	16.41	1.69 to 31.13	0.031
	Ineffective	−2.76	−18.25 to 12.73	0.708
Macro-level fidelity	Highly effective	6.95	2.15 to 11.75	0.007
	Ineffective	−1.24	−6.29 to 3.80	0.605
Meso-level fidelity	Highly effective	5.52	−0.66 to 11.71	0.076
	Ineffective	−0.75	−7.26 to 5.76	0.808
Micro-level fidelity	Highly effective	3.96	−3.24 to 11.15	0.257
	Ineffective	−0.78	−8.35 to 6.79	0.828
CI = confidence interval.				

## Discussion

The challenge of implementing any complex intervention is ensuring it becomes routine practice throughout the organisation. This is often described as making it ‘the way we do things around here’ (Haines *et al.*, 2004; Proctor *et al.*, 2011). Both THRIVE and i-THRIVE are frequently mentioned in CYPMH policy documents, by NHS England’s regional transformation boards, and in media on CYPMH. It is encouraging to note that over 70% of staff at comparison sites were aware of THRIVE, and nearly 23% exhibited perfect knowledge of THRIVE principles. At the site level, respondents from most i-THRIVE sites had an increased odds of reporting site implementation of THRIVE, compared to the average among comparison sites, though two comparison sites (Sites J and K) also had an increased odds of reporting site implementation of THRIVE. This motivated a sensitivity analysis in which Site J was excluded from the fidelity analysis; the results were not affected. When asked about personal use of THRIVE principles, there was no difference in the odds of respondents from many i-THRIVE sites compared to the average among comparison sites. Overall, staff at i-THRIVE sites were significantly more likely to be familiar with, understand, and apply THRIVE principles in their daily work. This indicates that the NIP aids in embedding THRIVE principles within an organisation.

Previous research has found staff development and training are key to successful implementation (Rahman *et al.*, 2012; Resnick *et al.*, 2018). Training activities and attendance at both i-THRIVE Academy and Community of Practice events were reported among implementation sites, and staff from implementation sites exhibited knowledge of THRIVE principles. Continued engagement of staff will likely be important for THRIVE integration, and formal assessments or competency checks may be useful to monitor delivery (Sanders *et al.*, 2022). In addition to educational activities, several of the NIP’s implementation strategies, including site diagnostics activities, coaching and support, and involving key partners were found to have positive results in a recent review (Ashcraft *et al.*, 2024).

Implementation research generates critical evidence for selecting effective strategies for health system transformation (Ashcraft *et al.*, 2024). Like the NIP, most implementations include multiple strategies (Ashcraft *et al.*, 2024), meaning it is difficult to attribute a successful implementation to any single strategy. There are few examples of implementation research for mental health, many of which focus on implementation effectiveness (Bartels *et al.*, 2022; Chung *et al.*, 2015, 2014; Kilbourne *et al.*, 2014; Morton *et al.*, 2020; Ruud *et al.*, 2021; Sinnema *et al.*, 2015; Toropova *et al.*, 2022; Waxmonsky *et al.*, 2014; Wells *et al.*, 2013) with a minority examining the implementation process more broadly (Bauer *et al.*, 2019; Leone *et al.*, 2022; Williams *et al.*, 2017). Among studies of the implementation process or implementation fidelity, a facilitator-implemented collaborative care model resulted in some improvements to team function; adoption of collaborative care model processes varied widely by site (Bauer *et al.*, 2019). Another study examined mediating factors for clinician adoption of evidence-based practices following an organisation-supported implementation strategy, reporting high fidelity to the strategy at both clinician and organisation levels (Williams *et al.*, 2017). Although implementation studies would benefit from enrolment of many participating sites to reduce the impact of between-site variation, additional site enrolment presents many logistical challenges.

Integrated care models like THRIVE are used in Europe, Australia and North America, and have been tailored for use in services for CYP (Hodgins *et al.*, 2024). Common features of integrated care models for youth include multidisciplinary staff members able to partner with external organisations and managers committed to integration, joint planning, and stakeholder partnership (Hodgins *et al.*, 2024). The THRIVE Framework targets a local community and relies on the involvement of multiple agencies to transform how these agencies provide mental healthcare for CYP (Wolpert *et al.*, 2019). Many of the implementation sites hosted events to build stakeholder relationships. We found that multi-agency cooperation was critical to the implementation of the NIP itself: the strength of working relationships in the local system moderated the effect of the NIP on THRIVE fidelity among sites. For future users of the NIP, an evaluation of local working relationships or pre-implementation efforts to engage with community stakeholders and strengthen these relationships would be worthwhile.

On average, the NIP had a modest impact on THRIVE fidelity among sites, without reaching the level of a statistically significant change. With a small number of sites included, the study may have been under-powered, and evaluation took place in the early years of a long-term programme—it is likely that the full impact of implementation efforts had not yet been realised. When implementation studies find a null effect, it is difficult to know if the effect is truly null or if the implementation itself was incorrect (Sanders *et al.*, 2022). Site-level changes in THRIVE fidelity had high variability; even when an implementation is supported in the same way for all sites, variation can occur and implementation success may depend on site characteristics (Augustsson *et al.*, 2015). To better understand the NIP impacts, we examined the moderating effect of an important site characteristic: the strength of local working relationships, finding that the NIP improved THRIVE fidelity (overall and at macro level) at implementation sites with strong local working relationships, compared to comparison sites with strong local working relationships. A similar result was found in a study of an organisational-level intervention for occupational health, where the implementation worked the best among units with strong collaboration (Augustsson *et al.*, 2015). To explain these results, the authors suggest that the intervention was a better fit for those units or that the units were more capable in adapting the intervention. Preliminary work can prepare sites for an implementation; for those sites interested in using the NIP, efforts to strengthen local working relationships prior to implementation should be considered.

The future direction of this research will involve comprehensive evaluations of the service and clinical impacts of the NIP. These evaluations will encompass a range of critical factors, including the accessibility and efficiency of services, clinical outcomes, patient experiences and the specific impacts on various sub-groups within the patient population. The latter will particularly focus on racial or ethnic minorities and distinct diagnostic categories, ensuring a broad and inclusive understanding of the NIP’s effectiveness.

## Limitations

There are several limitations to consider in this work. For the staff survey, the response rate was low (28.5%) compared to those in other implementation studies (46.8–83.1%) (Leone *et al.*, 2022; Toropova *et al.*, 2022). We would expect this to bias our results if the response rate was differential by implementation/comparison site groups, but there was no difference in response rate by these groups. The low response rate may indicate that the survey results are not generalisable.

When introducing new health policies, understanding their impact and identifying the contexts in which they are most effective is crucial. Estimating the mean effect presents several methodological challenges, especially for short pre-implementation periods or when few sites are involved. Unlike randomised controlled trials, the adoption of health policies is not random. This means that the characteristics of the implementation and control sites are likely to vary, leading to potential selection bias. It is often unclear which characteristics influence a site’s decision to adopt a health policy. Even with numerous characteristics measured, careful consideration of each characteristic’s role is necessary. Confounding is another potential bias, where some characteristics may influence both the decision to implement and the site’s capacity to do so effectively.

To adjust for these biases, various methods are available, but these methods can themselves introduce bias. Sensitivity analyses are essential in gaining a deeper understanding. The four-group propensity-score weighting DiD method is designed to mitigate potential selection biases and confounding factors (Stuart *et al.*, 2014). However, a key assumption of DiD, the parallel trends assumption, is not verifiable. Violations of the parallel trends assumption can lead to issues with time-varying confounding (Stuart *et al.*, 2014), complicating the interpretation of results. Thus, while our study provides valuable insights into the effectiveness of the NIP, these issues must be considered when interpreting the findings.

The variable transformation approaches used by comparison sites could be considered a limitation, as this complicates the interpretation of results. Strictly speaking, a comparison group where all sites were transforming their CAMHS into the THRIVE Framework would allow us to test the implementation strategies of the NIP specifically (i.e. all sites seeking to fit the THRIVE Framework but the implementation group testing the NIP implementation strategies). Four comparison sites reported using THRIVE as their transformation model, but this sample size is too small for a full analysis. The comparison group simply represents routine implementation, a common approach for implementation control groups (Smith and Hasan, 2020).

## Conclusions

This study’s investigation on NIP effectiveness in England offers significant insights into the implementation of complex health interventions, particularly in CAMHS. The findings underscore the importance of effective working relationships among local systems in the successful adoption and implementation of multi-agency health policies like i-THRIVE. Specifically, the study demonstrates that sites with highly effective working relationships exhibit substantial improvements in adhering to THRIVE principles, as evidenced by the increase in fidelity scores.

The broad awareness of the THRIVE framework among staff, even in comparison sites, highlights the programme’s permeation in the field of CYPMH. However, implementation strategies are critical to deeply embedding these principles within organisations. This distinction is crucial for policymakers and healthcare leaders aiming to foster more effective, integrated mental health services tailored to the needs of CYP.

Methodologically, the study navigates the challenges of evaluating health policy implementations in non-randomised settings. The use of four-group propensity-score-weighted DiD analysis is an effective approach to address potential biases, such as selection bias and confounding factors. It highlights the inherent complexities and limitations in evaluating policy impact in real-world settings. The study’s sensitivity analyses further strengthen the validity of its findings.

In conclusion, the NIP presents a promising model for CAMHS. Its emphasis on effective working relationships and the tailored approach to implementation are key factors in its success. The insights from this study contribute valuable knowledge to the ongoing efforts to improve CAMHS and can guide future policies and programmes aimed at enhancing the well-being of CYP.

## Supporting information

Sippy et al. supplementary material 1 Sippy et al. supplementary material

Sippy et al. supplementary material 2 Sippy et al. supplementary material

## Data Availability

All datasets and code are accessible on GitHub (Sippy, 2023).

## Supplementary material

The supplementary material for this article can be found at https://doi.org/10.1017/S2045796025000101.
