# Supplementary material for: Effect of a needs-based model of care on the characteristics of healthcare services in England: the i-THRIVE National Implementation Programme
Source: Epidemiol Psychiatr Sci. 2025 Mar 26;34:e21. doi: 10.1017/S2045796025000101 (PMC11955426; doi:10.1017/S2045796025000101)
Supplement: Sippy et al. supplementary material 1 — Sippy et al. supplementary material [file S2045796025000101sup001.docx]

Contents

[Supplemental Material S1: Delivery of i-THRIVE 2](#_Toc184732687)

[Supplemental Material S2: Additional Methodological Details 7](#_Toc184732688)

[Supplemental Material S3: Survey for CAMHS Staff 10](#_Toc184732689)

[Supplemental Material S4: Survey for Implementation Leads 12](#_Toc184732690)

[Supplemental Material S5: Survey for CAMHS Transformation Leads 19](#_Toc184732691)

[Supplemental Material S6: Sensitivity Analyses 26](#_Toc184732692)

[Supplemental Material S7: Additional Results 28](#_Toc184732693)

[Supplemental Material S8: i-THRIVE Effect Moderation 34](#_Toc184732694)

[References 35](#_Toc184732695)

## Supplemental Material S1: Delivery of i-THRIVE

**National i-THRIVE Programme**

The implementation mode for i-THRIVE l is fully described in the study protocol (Moore *et al.* 2023). Briefly, the National i-THRIVE Programme translates the macro-, meso-, and micro-level principles of THRIVE into six components to aid the implementation process. Brief descriptions of each component are in Table S1. Sites use the i-THRIVE Approach to Implementation, which has four phases, described in Table S2.

| Table S1 National i-THRIVE Programme Components | |
| --- | --- |
| i-THRIVE Component | **Description** |
| *i-THRIVE Community of Practice* | All implementing sites can participate in shared learning events, where active sites can share their implementation process and support one another |
| *i-THRIVE Implementation Support Team* | This team oversees the i-THRIVE Programme, Community of Practice, and Academy, and developed the Toolkit. Local services receive focused support from the team |
| *i-THRIVE Approach to Implementation* | This is a manual describing the four-phase, structured approach with definitions for integration and recommended evaluations |
| *i-THRIVE Toolkit* | This is a resource to support sites in fidelity to the implementation protocol but is flexible to the needs of the local context |
| *i-THRIVE Academy* | This is a series of coaching and training in the THRIVE principles targeting the frontline staff |
| *i-THRIVE Option Grids* | These are aids for decision-making to help staff, families, and CYP have productive conversations regarding care |

| Table S2 Phases of i-THRIVE Approach to Implementation | |
| --- | --- |
| Phase | **Description** |
| *One* | Comprehensive understanding of local systems and population needs |
| *Two* | Identifying training needs of staff and building system capacity |
| *Three* | Implementing the new system and establishing information infrastructures |
| *Four* | Learning, embedding, and sustaining changes to the system |

**Implementation Site Progress**

Annual progress reports were published in May 2017 and May 2018, and included updates from the implementation sites (National i-THRIVE Programme Team 2017). In addition, the i-THRIVE website includes summaries for each implementation site (i-THRIVE Team n.d.). Key achievements for each site are summarised in Table S3.

| Table S3: i-THRIVE Progress | |  |
| --- | --- | --- |
| Site | **Achievements** | |
| Site B | - Hosted a system-wide engagement event with over 100 participants - Mapped the needs and demands of the area - Collaboration with partner agencies and stakeholders | |
| Site C | - Completed an in-depth analysis of system services - Developed an app fo children and young people to better communicate with their clinicians and follow their progress - Offered additional support for those with long-term health conditions - Hosted a THRIVE clinic | |
| Site G | - Established an integrated partnership to focus on i-THRIVE - Mapped the care pathways of Site G - Established a group to focus on mental health and emotional wellbeing in schools | |
| Site L | - Engaged with third-sector organisations to employ THRIVE practitioners supporting CAMHS - Developed integrated access and care pathways - Staff attended i-THRIVE Academy - Used a radio programme to support children and young people - Offered additional support for those with eating disorders | |
| Sites D & M | - Programme of staff training events - Clinician attendance at i-THRIVE Academy - THRIVE needs-based groupings included in case records - Developed an in-school resilience programme | |
| Site O | - Focused on engagement with schools - Hosted a multi-agency engagement event with the i-THRIVE Implementation Support Team - Developed multi-disciplinary early help teams to support children and young people | |
| Site Q | - Implementation of multi-agency local agreements for collective accountability - Integration of THRIVE needs-based grouping into the clinical record system - Provided services in schools - Integrated THRIVE needs-based groupings into care plans | |
| Site R | - Developed a website for children, young people, and their families - Adoption of THRIVE by the local authority - Full review of CAMHS system with the i-THRIVE Implementation Support Team | |
| Site T | - Developed a website and services tailored to local children and young people - Clinicians trained in shared decision making (i-THRIVE Academy) | |

**Implementation Leads Self-Reported Experience**

As reported in the main manuscript, eight programme managers from seven implementation sites provided feedback on their site’s experience using i-THRIVE as part of the survey. The transformation leads survey responses were from NIP sites (Sites D, G, L (two managers), M, O, Q, and R). Responses related the planning of i-THRIVE are reported in Supplementary Figures 1 & 2. Responses related to the delivery of i-THRIVE are reported in Supplementary Figures 3 & 4.

| **Supplementary Figure 1** Planning of i-THRIVE |
| --- |
| 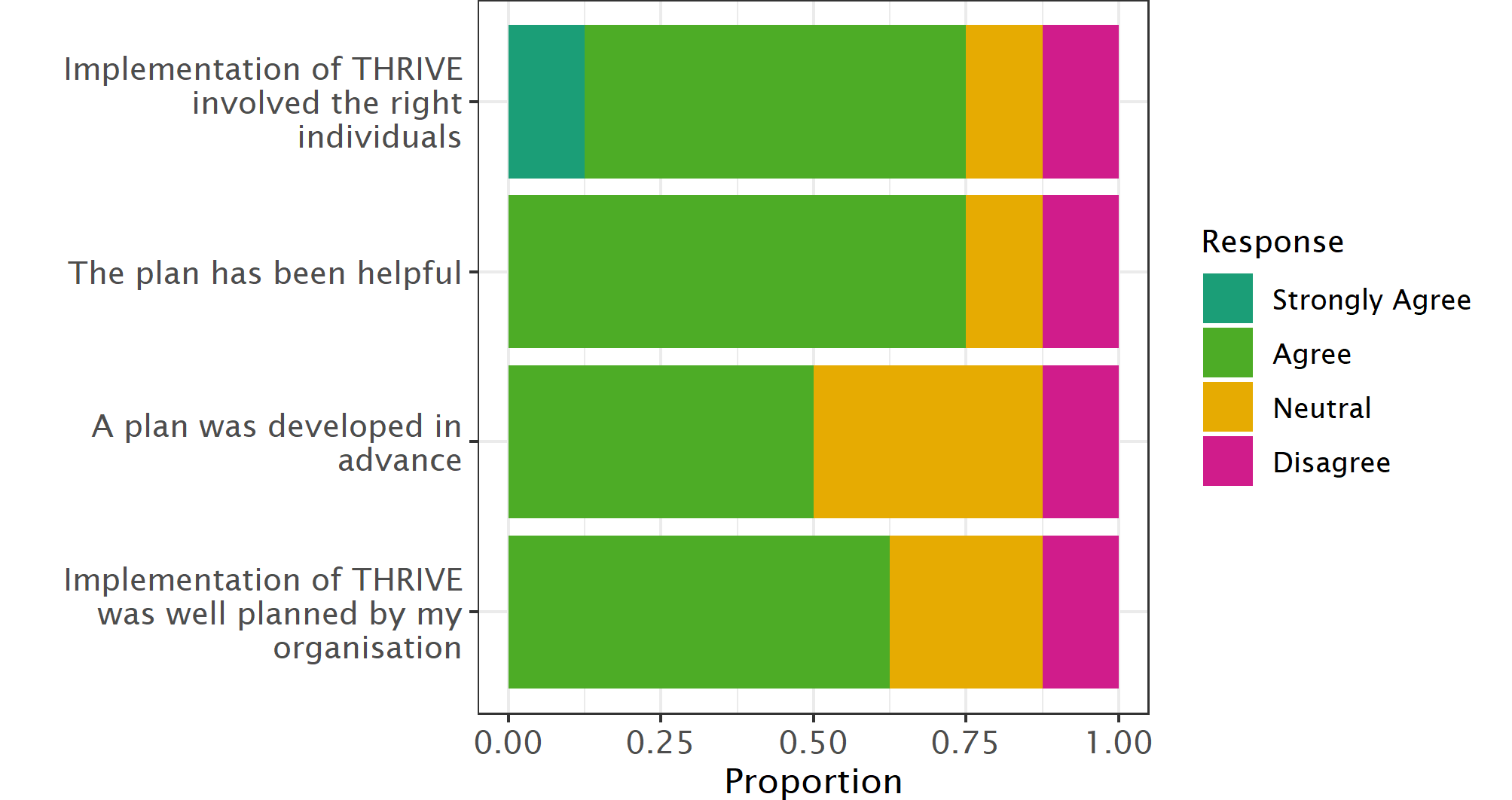 |
|  |
| \| **Supplementary Figure 2** Individuals Involved in i-THRIVE Planning \| \| --- \| \| 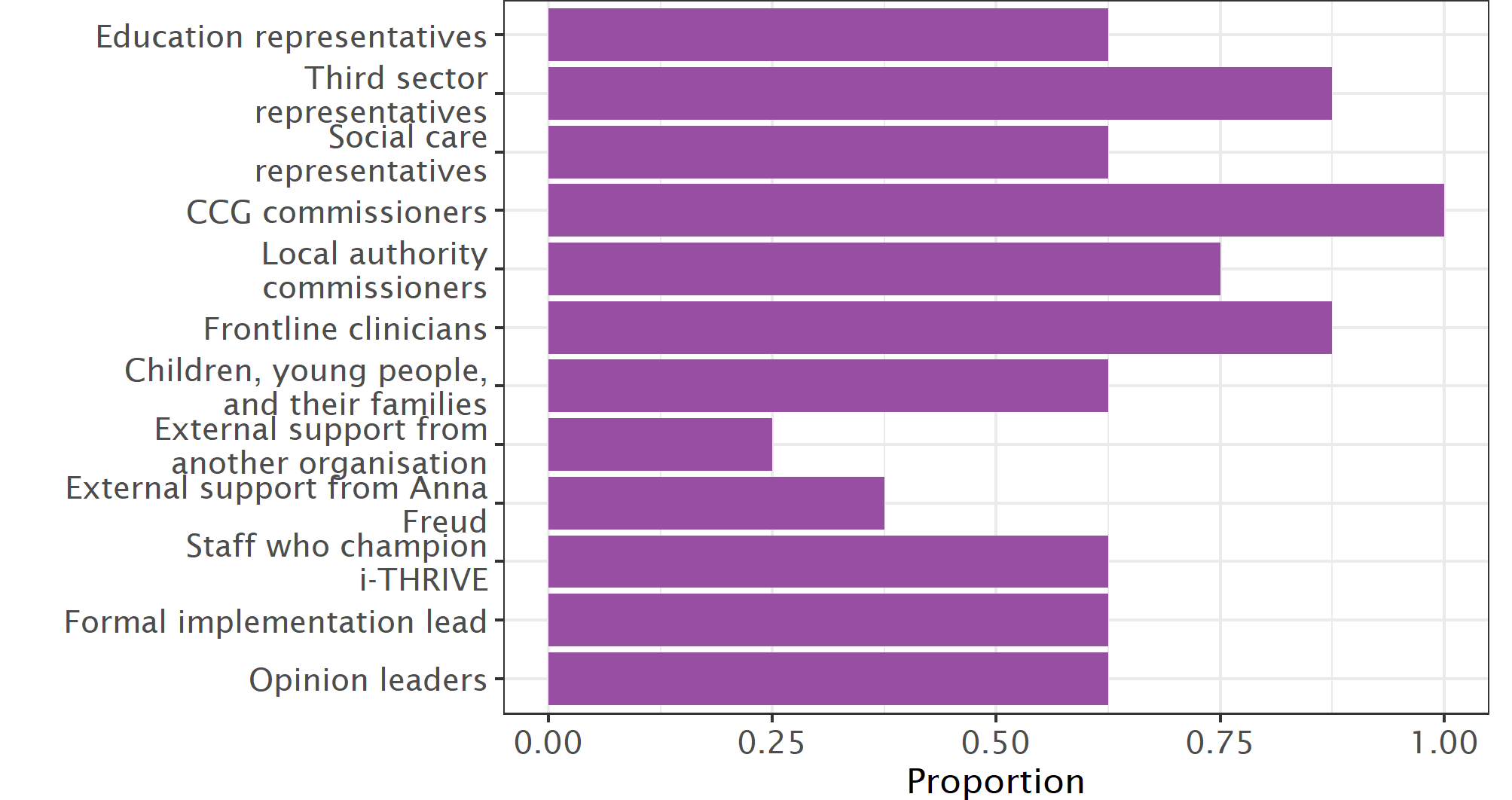 \| |
|  |

| **Supplementary Figure 3** Programme Managers Perspectives of i-THRIVE Delivery  There were two non-responses to the third and fourth questions**.** |
| --- |
| 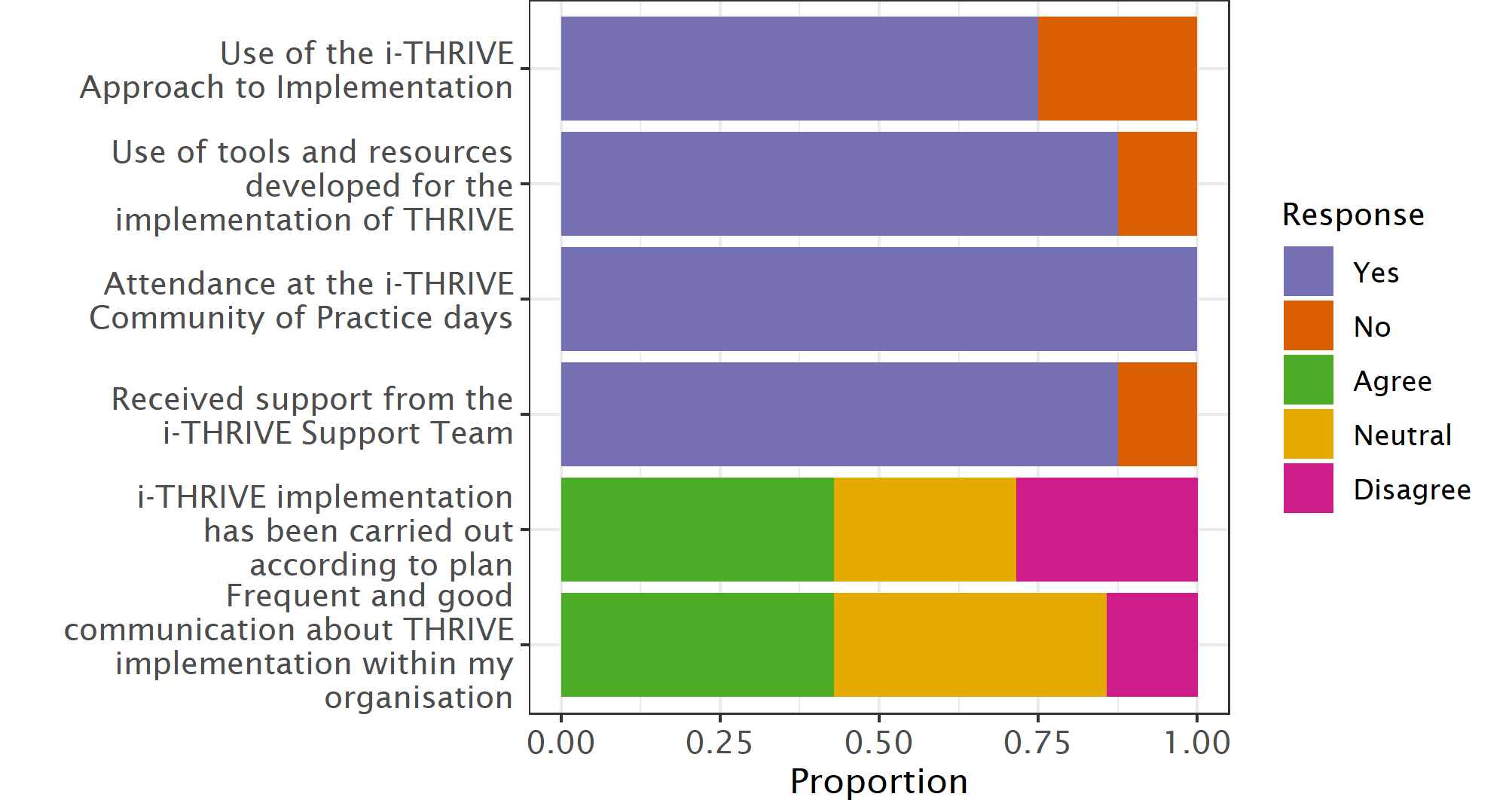 |
|  |
| **Supplementary Figure S4** Individuals Involved in Implementation  One site (R) did not respond to this series of questions, so the proportion is calculated from seven sites. There was also one non-response for the first and fourth questions. |
| 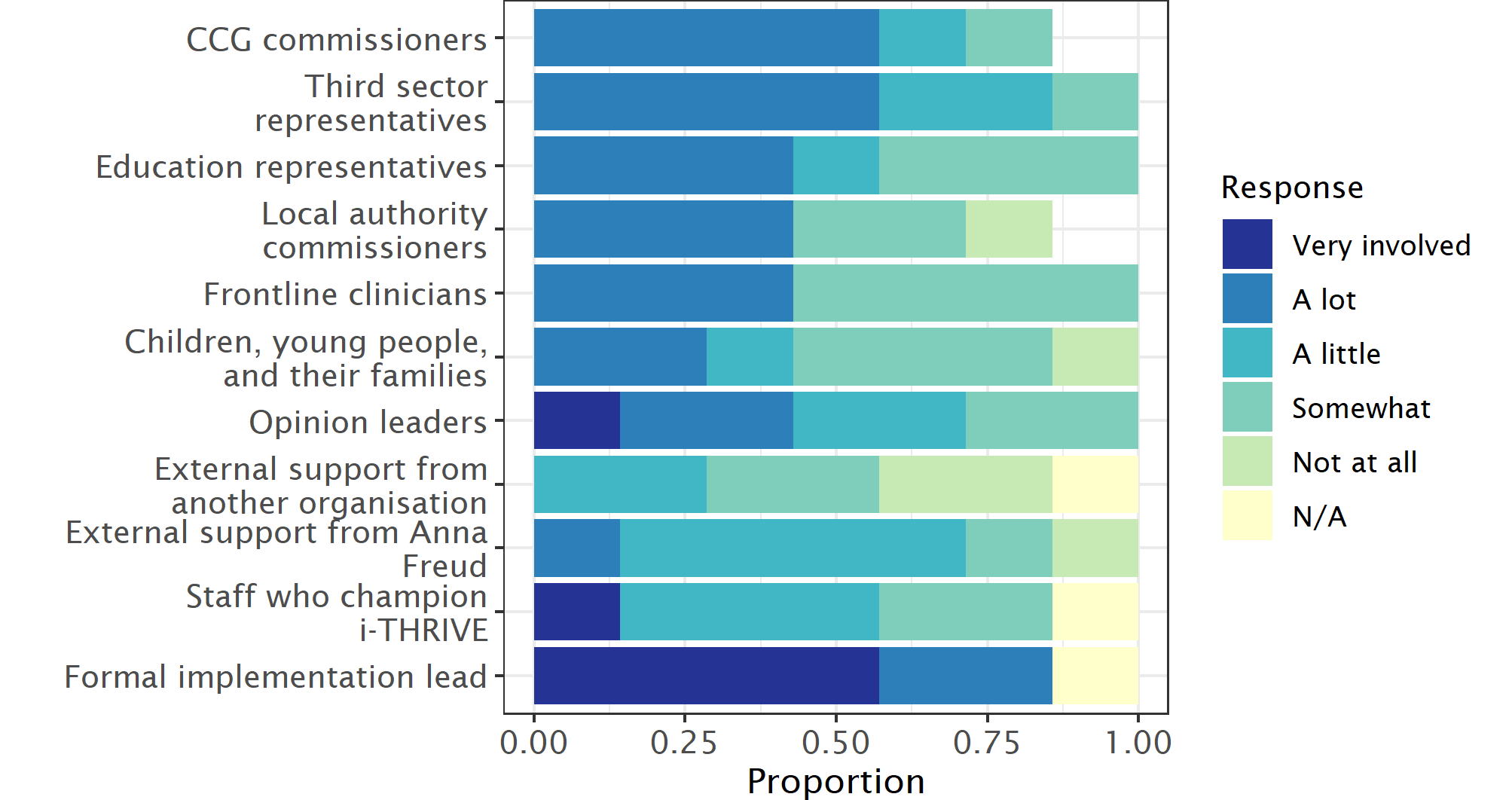 |

Most programme managers agreed that i-THRIVE was well-planned by their organisations, that the planning was helpful, and that the planning process involved the right individuals. Only half of the managers reported that the plan had been developed in advance. Many individuals were involved in the planning process, with most programme managers reporting planning involvement by those within the organisation at multiple levels (leaders and staff), individuals from other agencies (education, social care, local authorities, CCGs, and third sector), and importantly, five sites included children, young people, and their families in the planning process. There was less reliance on individuals from external organisations for planning.

Most programme managers reported used of the i-THRIVE Approach to Implementation (all except Sites M and Q), using tools and resources (all except Site G), and receiving support from the i-THRIVE Support Team (all except Site Q). All programme managers reported attendance at the i-THRIVE Community of Practice events. Two programme managers disagreed with the statement “*i-THRIVE implementation has been carried out according to plan*” (Sites G and L). There were two programme managers surveyed from Site L; the other programme manager for this site agreed with the statement. The Site G programme manager also disagreed with the statement “*There is frequent and good communication about how THRIVE implementation is going within my organisation*”. More than half of programme managers reported that formal implementation leads, third sector representatives, and CCG commissioners were “very involved” or had “a lot” of involvement in implementation.

## Supplemental Material S2: Additional Methodological Details

This section of the supplemental materials provides further details of the methods used for our study.

**Study Setting and Design**

Most sites were equivalent to a single CCG, but five included multiple CCGs (see Table S1). There were administrative changes during the study in April 2017: Central Manchester, North Manchester, and South Manchester CCGs merged to form the Manchester CCG, eight GP practices moved from South Norfolk CCG to Norwich CCG, and one GP practice transitioned from Bradford City to Bradford Districts (NHS England 2019b).

| **Sites** | | **CCGs** |
| --- | --- | --- |
| i-THRIVE | Bexley | - |
|  | Cambridgeshire and Peterborough | - |
|  | Camden | - |
|  | Hertfordshire | 1. East and North Hertfordshire 2. Herts Valley |
|  | Luton | - |
|  | Manchester | 1. Central Manchester 2. North Manchester 3. Salford 4. South Manchester |
|  | Stockport | - |
|  | Tower Hamlets | - |
|  | Waltham Forest | - |
|  | Warrington | - |
| Comparison | Bradford | 1. Airedale, Wharfdale and Craven 2. Bradford City 3. Bradford Districts |
|  | Ipswich and East Suffolk | - |
|  | Lewisham | - |
|  | Norfolk | 1. Great Yarmouth and Waveney 2. North Norfolk 3. Norwich 4. South Norfolk 5. West Norfolk |
|  | Northampton | 1. Corby 2. Nene |
|  | Portsmouth | - |
|  | Southampton | - |
|  | Stoke-on-Trent | - |
|  | Sunderland | - |
|  | South Worcestershire | - |

**Surveys**

In the staff survey, staff were asked to identify five THRIVE needs categories correctly, to evaluate knowledge of the THRIVE Framework. As part of a validation process, fifty individuals not involved in the evaluation took this assessment. Those familiar with THRIVE showed a 75% or higher accuracy rate, while those unfamiliar scored 40% or lower. This result suggests the questions effectively measured specific knowledge related to the programme rather than general tendencies or biases. Site leaders distributed the survey to professionals involved in providing and commissioning CYPMH services at both i-THRIVE and comparison sites. The survey was administered in August 2019, October 2019, and January 2020.

The transformation leads survey responses were from NIP sites (Sites D, G, L (two managers), M, O, Q and R) as well as comparison sites (Sites A, E, I, J (two managers), K, P, and S). These surveys were distributed from January to May 2020.

**Fidelity Ratings**

Fidelity scoring was based on information collected during interviews using the i-THRIVE Assessment Tool and quantitative data on service use collected from the NHS. Interviewees were drawn from multiple agencies, including CAMHS, CCGs, education, local authorities, and third sector. Purposive sampling was used to recruit three staff members at macro (senior leadership), meso (service management), and micro (frontline staff) levels for in-depth interviews. Interviews were conducted over the phone and were recorded, lasting from 45 minutes to 1 hour. All twenty sites received a fidelity score (1: low, 4: high) for each of 75 THRIVE principles, before (between December 2017 and July 2019) and after implementation (between October 2018 and February 2020), resulting in 40 sets of scores. The median interval period between baseline and follow-up scoring was 178 days. A total of 352 interviews were conducted (182 baseline interviews and 170 follow-up interviews). At least two independent raters scored for each set. To determine the interrater reliability among the raters of a set, Krippendorff’s alpha was calculated (Hayes and Krippendorff 2007). This metric ranges from 0 (low) to 1 (high) and is appropriate for two or more raters providing ordinal scores. Rater scores were averaged for each site and period. When there was not enough information to assign a score, we estimated the missing scores using the mean difference between pre- and post-intervention scores for the site, based on the score for the available period. The i-THRIVE Assessment Tool has not been validated.

**Auxiliary Data**

Site characteristics were measured in 2016 and 2019, unless stated otherwise. They included population density (total population per square kilometre) for each CCG (Office for National Statistics 2016, 2017), annual funding support (£100,000 increments) per CCG (NHS England 2017), Indices of Multiple Deprivation (IMD) ranks (Department for Communities and Local Government 2015, 2019), the initial number of CCGs per site (NHS England 2019a), and compliance in 2017 with the CCG Improvement and Assessment Framework Mental Health Transformation Milestones (NHS England 2017). For sites comprising multiple CCGs, we summed the annual funding and averaged the population density, effectiveness of working relationships in the local system, and transformation compliance across CCGs. Site-level IMD ranks were calculated using the method recommended by the Office of National Statistics (Noble *et al.* 2019).

**Statistical Analyses**

All data visualisation, cleaning, and propensity score modelling was performed in R version 4.2.3 (The R Foundation for Statistical Computing, Vienna, Austria) and RStudio version 2023.03.0 (RStudio, Inc., Boston, MA, USA), with R packages cowplot, extrafont, ggpattern, ggspatial, gridExtra, haven, irr, nnet, RColorBrewer, readxl, reshape2, sf, tableone, and tidyverse (Auguie 2017; Chang 2023; Dunnington 2023; FC *et al.* 2022; Gamer *et al.* 2019; Neuwirth 2014; Pebesma 2018; Pebesma and Bivand 2023; Venables and Ripley 2002; Wickham 2007; Wickham *et al.* 2019; Wickham and Bryan 2023; Wickham and Miller 2017; Wilke 2020; Yoshida and Bartel 2022). The simple DiD, four-group weighted DiD, and effect modification analyses were performed in SAS® version 3.81 (SAS Institute Inc., Cary, NC, USA) in SAS® Studio.

## Supplemental Material S3: Survey for CAMHS Staff

1. Which of the following best describes your organisation?
   1. NHS
   2. CCG
   3. Local authority
   4. Third sector
   5. Education
   6. Other (please state)
2. Which of the following best describes the primary purpose of the service that you work in?
   1. Specialist mental health services
   2. Brief intervention/counselling or support
   3. A service that is aimed at preventing mental health problems in children
   4. Supporting children and young people in the community who are considered to be high risk
   5. Triage, signposting, advice or assessment
   6. Children’s community services
   7. Social care
   8. Education
   9. Commissioning
   10. Admin
   11. Other (please state)
3. Which of the following best describes how you spend the majority of your time in your current role?
   1. Frontline practitioner with direct contact with children and young people
   2. Manager of a service or team
   3. Senior leadership within an organisation
   4. Commissioner
   5. Admin
   6. Other (please state)
4. Have you heard of the THRIVE Framework?
   1. Yes
   2. No
5. How have you heard of THRIVE? Please select all that apply.
   1. Communications from within my organisation (eg. newsletter, website, other communications to staff)
   2. Communications from outside my organisation (eg. local policy, reports from other organisations)
   3. Discussed during a training course, event or meeting
   4. In the media or online
   5. Talking to colleagues
   6. Talking to friends or family (not including colleagues)
6. Do you consider your service to be implementing THRIVE?
   1. Yes
   2. No
   3. Unsure
   4. (Optional) If you are unsure, could you provide some more information?
7. Do you personally feel that you use THRIVE principles in your daily practice?
   1. Yes
   2. No
   3. Unsure
   4. (Optional) If so, in what ways do you use THRIVE in your daily practice?
8. To your knowledge, is there a quality improvement initiative focused on implementing THRIVE in your organisation? This could be in a different service than the one where you work.
   1. Yes
   2. No
   3. (Optional) Do you know what aspect of the service the QI initiative is focused on improving?
9. Which of the following are THRIVE groups? Please select all that apply.
   1. Getting advice and signposting
   2. Getting more help
   3. Getting crisis support
   4. Getting help
   5. Getting risk support
   6. Getting specialist services

## Supplemental Material S4: Survey for Implementation Leads

We'd like to hear your views about the implementation of the THRIVE Framework in your area. Your responses are important for us to understand the factors that contribute to its implementation.

We thank you for your help!

For any questions, please contact Liz Simes, Research Manager, at e.simes@ucl.ac.uk. More information is available on the i-THRIVE website.

1. Which of the following best describes your organisation?
   1. NHS
   2. CCG
   3. Local authority
   4. Third sector
   5. Education
   6. Other (please state)
2. Please select the locality you work in.
3. Which of the following best describes the primary purpose of the service that you work in?
   1. Specialist mental health services
   2. Brief intervention/counselling or support
   3. A service that is aimed at preventing mental health problems in children
   4. Supporting children and young people in the community who are considered to be high risk
   5. Triage, signposting, advice or assessment
   6. Other (please state)
4. Please provide the name of your organisation.
5. Which of the following best describes how you spend the majority of your time in your current role?
   1. Frontline practitioner with direct contact with children and young people
   2. Manager of a service or team
   3. Senior leadership within an organisation
   4. Commissioner
   5. Other (please state)
6. How long have you been employed by your organisation?
   1. Less than 1 year
   2. 1 - 5 years
   3. 5 – 10 years
   4. 10 – 15 years
   5. 15 – 20 years
   6. Over 20 years
7. Have you been involved in the implementation of THRIVE in your locality?
   1. Yes
   2. No
8. Is your organisation implementing THRIVE?
   1. Yes
   2. No
   3. To some extent
   4. (Optional) If you have ticked ‘to some extent’, could you provide a brief explanation of this answer?
9. Please select the extent to which you agree with the following statements around planning implementation.

|  | Strongly agree | Agree | Neither agree nor disagree | Disagree | Strongly disagree |
| --- | --- | --- | --- | --- | --- |
| A plan for implementing THRIVE was developed in advance |  |  |  |  |  |
| The implementation plan has been helpful |  |  |  |  |  |
| Implementation of THRIVE involved the right individuals |  |  |  |  |  |
| Planning of the implementation of THRIVE was well done by my organisation |  |  |  |  |  |

1. Who was involved in developing a plan to implement THRIVE? Please select all that apply.
   1. Opinion leaders - active and respected voices within the service
   2. Formally appointed i-THRIVE implementation lead
   3. Staff who champion i-THRIVE in addition to their regular duties
   4. External implementation support from the Anna Freud Centre
   5. External implementation support from another external organisation (eg. NHS Improvement)
   6. Children, young people and their families
   7. Frontline clinicians
   8. Commissioners from the local authority
   9. Commissioners from the CCG
   10. Representatives from social care
   11. Representatives from the third sector
   12. Representatives from education
   13. Other (please specify)
2. Did you use the “i-THRIVE Approach to Implementation” to help guide your approach?*

* The "i-THRIVE Apporach to Implementation" has been developed by the Anna Freud Centre and the Tavistock and Portman NHS

Foundation Trust and is designed to guide services through the process of implementing THRIVE.

- 1. Yes
  2. No
  3. I don’t know
  4. To some extent
  5. (Optional) If you have ticked ‘to some extent’ would you please provide a brief explanation of this answer.

1. Please answer the following questions about your use of the implementation support by the National i-THRIVE implementation Team based at the Anna Freud Centre and the Tavistock and Portman NHS Foundation Trust.

|  | Yes | No |
| --- | --- | --- |
| Did you use the "i-THRIVE Approach to Implementation" to help guide your approach? |  |  |
| Did you use any of the implementation tools developed for i-THRIVE implementation (available online or through the i-THRIVE Programme?) |  |  |
| Did you attend any of the i-THRIVE Community of Practice days? |  |  |
| Did you receive support from the i-THRIVE Implementation Support Team at the Anna Freud Centre or the Tavistock and Portman NHS Foundation Trust? |  |  |

1. Please add any further comments about the i-THRIVE Implementation Programme.
2. Did you use any other implementation approaches to help guide your approach?
   1. Yes
   2. No
3. If yes, was this a structured approach to implementation?
   1. Yes
   2. No
   3. If yes, please provide further details.
4. To what extent do you agree with the following statements?

|  | Strongly agree | Agree | Neither agree nor disagree | Disagree | Strongly disagree |
| --- | --- | --- | --- | --- | --- |
| Some of our staff have become i-THRIVE champions, actively supporting and promoting THRIVE implementation beyond what is required |  |  |  |  |  |
| Clinical staff take an active interest in i-THRIVE related problems and successes |  |  |  |  |  |
| Managers in the service actively support i-THRIVE implementation |  |  |  |  |  |
| Commissioners actively support the implementation of i-THRIVE |  |  |  |  |  |
| Other agencies in the local system actively  support i-THRIVE implementation |  |  |  |  |  |

1. To what extent were the following people involved in implementing THRIVE?

|  | Strongly agree | Agree | Neither agree nor disagree | Disagree | Strongly disagree |
| --- | --- | --- | --- | --- | --- |
| Formally appointed i-  THRIVE implementation  lead |  |  |  |  |  |
| Staff who champion i-  THRIVE in addition to  their regular duties |  |  |  |  |  |
| External implementation  support from the Anna Freud Centre or the  Tavistock and Portman NHS Foundation Trust |  |  |  |  |  |
| External implementation  support from another  organisation (eg. NHS  Improvement) |  |  |  |  |  |
| Opinion leaders - active  and respected voices in the service |  |  |  |  |  |
| Children, young people  and their families |  |  |  |  |  |
| Frontline clinicians |  |  |  |  |  |
| Local authority or social care representatives |  |  |  |  |  |
| Education representatives |  |  |  |  |  |
| Third sector representatives |  |  |  |  |  |
| CCG representatives |  |  |  |  |  |

1. Do you feel the correct people were involved in carrying out implementation? If not, who should have been?
2. To what extent do you agree with the following statements?

|  | Strongly agree | Agree | Neither agree nor disagree | Disagree | Strongly disagree |
| --- | --- | --- | --- | --- | --- |
| My organisation works well with the local authority to meet children and young  people's mental health  needs in my area |  |  |  |  |  |
| My organisation works well with schools to meet children and young  people's mental health  needs in my area |  |  |  |  |  |
| My organisation works well with the voluntary  sector to meet children and young people's  mental health needs in my area |  |  |  |  |  |
| My organisation works well with the NHS to  meet children and young  people's mental health  needs in my area |  |  |  |  |  |
| Health and social care  commissioners work well together in my area |  |  |  |  |  |
| My organisation  generally has good relationships with other  agencies in my area |  |  |  |  |  |
| The right organisations  were involved in the  implementation of THRIVE |  |  |  |  |  |

(Optional) Please provide any further comments.

1. To what extent do you agree with the following statements?

|  | Strongly agree | Agree | Neither agree nor disagree | Disagree | Strongly disagree |
| --- | --- | --- | --- | --- | --- |
| i-THRIVE  implementation has been carried out  according to plan to date |  |  |  |  |  |
| There is frequent and  good communication  about how THRIVE  implementation is going within my organisation |  |  |  |  |  |
| Data is used to guide i-THRIVE implementation  within my organisation |  |  |  |  |  |

(Optional) Please provide any further comments.

21.Are you happy to be contacted for further details about this survey? If yes, please add your email below.

## Supplemental Material S5: Survey for CAMHS Transformation Leads

We'd like to hear your views about the transformation of the mental health services for children and young people in your area. Your responses are important for us to understand the factors that can impact the CAMHS re-design locally.

We thank you for your help!

For any questions, please contact Liz Simes, Research Manager, at e.simes@ucl.ac.uk. More information is available on the i-THRIVE website.

1. Which of the following best describes your organisation?
   1. NHS
   2. CCG
   3. Local authority
   4. Third sector
   5. Education
   6. Other (please state)
2. Please select the locality you work in.
3. Which of the following best describes the primary purpose of the service that you work in?
   1. Specialist mental health services
   2. Brief intervention/counselling or support
   3. A service that is aimed at preventing mental health problems in children
   4. Supporting children and young people in the community who are considered to be high risk
   5. Triage, signposting, advice or assessment
   6. Other (please state)
4. Please provide the name of your organisation.
5. Which of the following best describes how you spend the majority of your time in your current role?
   1. Frontline practitioner with direct contact with children and young people
   2. Manager of a service or team
   3. Senior leadership within an organisation
   4. Commissioner
   5. Other (please state)
6. How long have you been employed by your organisation?
   1. Less than 1 year
   2. 1 - 5 years
   3. 5 – 10 years
   4. 10 – 15 years
   5. 15 – 20 years
   6. Over 20 years
7. Have you been involved in the CAMHS transformation in your locality?
   1. Yes
   2. No
8. Does your organisation implement the CAMHS transformation plan developed by local commissioners?
   1. Yes
   2. No
   3. To some extent
   4. (Optional) If you have ticked ‘to some extent’, could you provide a brief explanation of this answer?
9. Please select the extent to which you agree with the following statements around planning implementation.

|  | Strongly agree | Agree | Neither agree nor disagree | Disagree | Strongly disagree |
| --- | --- | --- | --- | --- | --- |
| The implementation of CAMHS transformation  was well planned by my organisation |  |  |  |  |  |
| A plan for implementing CAMHS transformation was developed in advance |  |  |  |  |  |
| The implementation plan has been helpful |  |  |  |  |  |
| Implementing CAMHS transformation involved the right individuals |  |  |  |  |  |

1. Who was involved in developing a plan for transforming CAMHS? Please select all that apply.
   1. Opinion leaders - active and respected voices within the service
   2. Formally appointed CAMHS transformation lead
   3. Staff who champion CAMHS transformation in addition to their regular duties
   4. External implementation support from an external organisation (eg. research centre, university, NHS Improvement)
   5. Children, young people and their families
   6. Frontline clinicians
   7. Commissioners from the local authority
   8. Commissioners from the CCG
   9. Representatives from social care
   10. Representatives from the third sector
   11. Representatives from education
   12. Other (please specify)
2. Did you use a service model or approach to help you guide CAMHS transformation (e.g. CAPA, the THRIVE Framework)?
   1. Yes
   2. No
3. Did you use tools or resources to help you guide local CAMHS transformation?
   1. Yes
   2. No
   3. I don’t know
   4. To some extent
   5. (Optional) If you have ticked ‘to some extent’ would you please provide a brief explanation of this answer.
4. Did you receive support from external organisations (eg. research centre, university, NHS Improvement) to help you guide local CAMHS transformation?
   1. Yes
   2. No
   3. I don’t know
   4. To some extent
   5. (Optional) If you have ticked ‘to some extent’ would you please provide a brief explanation of this answer.
5. Did you attend any events that helped you with local CAMHS transformation?
   1. Yes
   2. No
   3. (Optional) If yes, would you please provide more details to your answer.
6. To what extent do you agree with the following statements?

|  | Strongly agree | Agree | Neither agree nor disagree | Disagree | Strongly disagree |
| --- | --- | --- | --- | --- | --- |
| Some of our staff have become champions for implementing CAMHS transformation, actively supporting and promoting it beyond what is required |  |  |  |  |  |
| Clinical staff take an active interest in the CAMHS transformation related problems and successes |  |  |  |  |  |
| Managers in the service actively support the implementation of the CAMHS transformation |  |  |  |  |  |
| Commissioners actively support the implementation of CAMHS transformation |  |  |  |  |  |
| Other agencies in the local system actively  support the implementation of CAMHS transformation |  |  |  |  |  |

1. To what extent were the following people involved in implementing CAMHS transformation?

|  | Strongly agree | Agree | Neither agree nor disagree | Disagree | Strongly disagree |
| --- | --- | --- | --- | --- | --- |
| Formally appointed CAMHS transformation  lead |  |  |  |  |  |
| Staff who champion CAMHS transformation in addition to  their regular duties |  |  |  |  |  |
| External implementation  support from expert  organisations (eg. NHS  Improvement, Anna Freud Centre) |  |  |  |  |  |
| Opinion leaders - active  and respected voices in  the service |  |  |  |  |  |
| Children, young people  and their families |  |  |  |  |  |
| Frontline clinicians |  |  |  |  |  |
| Local authority or social care representatives |  |  |  |  |  |
| Education representatives |  |  |  |  |  |
| Third sector representatives |  |  |  |  |  |
| CCG representatives |  |  |  |  |  |

1. Do you feel the correct people were involved in carrying out implementation? If not, who should have been?
2. To what extent do you agree with the following statements?

|  | Strongly agree | Agree | Neither agree nor disagree | Disagree | Strongly disagree |
| --- | --- | --- | --- | --- | --- |
| My organisation works well with the local authority to meet children and young  people's mental health  needs in my area |  |  |  |  |  |
| My organisation works well with schools to meet children and young  people's mental health  needs in my area |  |  |  |  |  |
| My organisation works well with the voluntary  sector to meet children and young people's  mental health needs in my area |  |  |  |  |  |
| My organisation works well with the NHS to  meet children and young  people's mental health  needs in my area |  |  |  |  |  |
| Health and social care  commissioners work well together in my area |  |  |  |  |  |
| My organisation  generally has good relationships with other  agencies in my area |  |  |  |  |  |
| The right organisations  were involved in the  implementation of CAMHS transformation |  |  |  |  |  |

(Optional) Please provide any further comments.

1. To what extent do you agree with the following statements?

|  | Strongly agree | Agree | Neither agree nor disagree | Disagree | Strongly disagree |
| --- | --- | --- | --- | --- | --- |
| The  implementation of CAMHS transformation has been carried out  according to plan to date |  |  |  |  |  |
| There is frequent and  good communication  about how the  implementation of CAMHS transformation is going  within my organisation |  |  |  |  |  |
| Data is used to guide the implementation of CAMHS transformation  within my organisation |  |  |  |  |  |

(Optional) Please provide any further comments.

21.Are you happy to be contacted for further details about this survey? If yes, please add your email below.

## Supplemental Material S6: Sensitivity Analyses

This section of the supplemental materials provides a detailed description of the sensitivity analyses conducted for our study. These analyses are crucial in validating the robustness of our findings and include the use of alternative methods for health policy analysis, different model specifications for effect estimation, and the consideration of excluding non-compliant control sites. Additionally, we present the results of these analyses together, facilitating a straightforward comparison.

**Alternative Method of Analysis**

For the estimation of the NIP effect, we employed two alternative methods: the standard difference in differences (DiD) and the four-group propensity score weighted DiD (Stuart *et al.* 2014). The standard DiD method, a staple in health policy evaluations, allows for a comparison between implementation and control units. This method determines the impact of the implementation by comparing the change in outcomes at control units before and after the implementation with the corresponding change at implementation units. We derived the standard DiD estimates using maximum-likelihood repeated measures linear regression, incorporating an auto-regressive correlation structure. This approach is advantageous due to its simplicity and directness, offering a clear view of the intervention's impact. The standard DiD results are in Table S1.

| **Table S1** Standard DiD Estimates of the Impact of the National i-THRIVE Programme on THRIVE Fidelity | | | |
| --- | --- | --- | --- |
| **Outcome** | **Estimate** | **95% CI** | **p-value** |
| Overall Fidelity | 7.88 | -3.15–18.92 | 0.150 |
| Macro-level Fidelity | 3.25 | -0.61–7.10 | 0.093 |
| Meso-level Fidelity | 1.59 | -3.23–6.41 | 0.496 |
| Micro-level Fidelity | 3.05 | -1.43–7.53 | 0.169 |
| CI = confidence interval, DiD = difference in differences | | | |

The four-group propensity score weighted DiD, on the other hand, offers a more nuanced analysis. It accounts for potential imbalances between the implementation and control groups by weighting their characteristics. This method enhances the accuracy of the estimates by reducing biases that could arise from unobserved confounding variables or selection biases inherent in non-randomised studies. By comparing these two methods, we aim to validate the consistency of our findings and ensure that our conclusions are not solely dependent on a single analytical approach. Such a comprehensive analytical strategy strengthens the reliability of our conclusions regarding the effectiveness of the NIP in improving mental health care services for children and young people in England.

**Alternative Model Specification**

We included effect estimates from a Gaussian-distributed identity-link generalised linear model using generalised estimating equations with an auto-regressive correlation structure, as well as a Gamma-distributed identity-link generalised linear model using generalised estimating equations with an auto-regressive correlation structure. These results are in Table S2. Although the effect estimates from the Gamma GEE model reach a level of statistical significance for meso-level fidelity, the model fit metric (QICu) does not support selection of this model over the Gaussian GEE.

| **Table S2** Estimates of the Impact of the National i-THRIVE Programme on THRIVE Fidelity | | | | | |
| --- | --- | --- | --- | --- | --- |
| **Outcome** | **Estimator** | **QICu** | **Estimate** | **95% CI** | **p-value** |
| Overall Fidelity | Gaussian GEE | 47.00 | 6.07 | -4.43–16.56 | 0.257 |
|  | Gamma GEE | 2421.88 | 8.92 | -0.05–17.89 | 0.051 |
| Macro-level Fidelity | Gaussian GEE | 47.00 | 2.56 | -1.19–6.30 | 0.180 |
|  | Gamma GEE | 2956.39 | 3.33 | -0.24–6.90 | 0.067 |
| Meso-level Fidelity | Gaussian GEE | 47.00 | 2.85 | -0.77–6.48 | 0.123 |
|  | Gamma GEE | 1622.20 | 5.04 | 2.29–7.78 | <0.001 |
| Micro-level Fidelity | Gaussian GEE | 47.00 | 0.66 | -5.13–6.45 | 0.823 |
|  | Gamma GEE | 1712.07 | 2.22 | -3.18–7.63 | 0.420 |
| CI = confidence interval, GEE = generalized estimating equations, QICu = quasi-Akaike information criterion (u) | | | | | |

**Exclusion of Non-Compliant Control Sites**

To assess the influence of non-compliant comparison sites, we repeated the analysis while excluding Site J, the comparison site with the strongest evidence for non-compliance. These results are provided in Table S3.

| **Table S3** Estimates of the Impact of the National i-THRIVE Programme on THRIVE Fidelity while Excluding Non-compliant Comparison Sites | | | |
| --- | --- | --- | --- |
| **Outcome** | **Estimate** | **95% CI** | **p-value** |
| Overall Fidelity | 5.31 | -6.46–17.07 | 0.352 |
| Macro-level Fidelity | 2.35 | -1.77–6.47 | 0.242 |
| Meso-level Fidelity | 2.18 | -2.74–7.09 | 0.360 |
| Micro-level Fidelity | 0.79 | -4.76–6.33 | 0.767 |
| CI = confidence interval | | | |

## Supplemental Material S7: Additional Results

**Surveys**

The staff survey was distributed to 2,415 staff. Responses included 261 staff (37.9%) were from i-THRIVE sites, while 428 respondents (62.1%) were from comparison sites. The mean response rate was 33.0% at i-THRIVE sites (95% CI: 16.3–49.7%) and 34.3% at comparison sites (95% CI: 20.6–48.0%). There was no significant difference in the response rate (p = 0.9). Demographic and professional characteristics of the respondents are summarised in Table S1.

| Table S1: Staff Survey Respondents | | | |
| --- | --- | --- | --- |
|  |  | i-THRIVE | Comparison |
|  | n  (%) | 261  (37.9) | 428  (62.1) |
| Organisation | NHS Site | 220  (84.6) | 409  (96.0) |
|  | Local authority | 15  (5.8) | 7  (1.6) |
|  | Third sector | 11  (4.2) | 5  (1.2) |
|  | Education | 7  (2.7) | 1  (0.2) |
|  | Missing | 1 | 2 |
| Position | Frontline practitioner | 181  (69.6) | 297  (69.9) |
|  | Service/team manager | 47  (18.1) | 50  (11.8) |
|  | Senior lead | 18  (6.9) | 21  (4.9) |
|  | Commissioner | 5  (1.9) | 2  (0.5) |
|  | Other | 9  (3.5) | 55  (12.9) |
|  | Missing | 1 | 3 |
| Service Provided | Specialist mental health | 170  (65.1) | 339  (80.3) |
|  | Brief intervention | 29  (11.1) | 18  (4.3) |
|  | Prevention | 14  (5.4) | 13  (3.1) |
|  | Risk support | 22  (8.4) | 32  (7.6) |
|  | Triage, advice, assessment | 9  (3.5) | 6  (1.4) |
|  | Other | 17  (6.5) | 14  (3.3) |
|  | Missing | 0 | 6 |

Consistent with THRIVE principles, staff from implementation sites were from a broader range of services, with fewer from traditional specialist CYPMH services (65.1% vs. 80.3%); more respondents provided brief intervention (11.1% vs. 4.3%), prevention (5.4% vs. 3.1%), risk support (8.4% vs. 7.6%), or dedicated triage/advice/assessment (3.5% vs. 1.4%). i-THRIVE site service profiles differed from comparison sites, indicating organisational change in line with CYP needs (p<0.0001, Cramer’s V = 0.19). We examined knowledge, attitudes and behaviours between implementation and comparison sites separately (Table S2). Although respondents were broadly aware of the THRIVE Framework, those from implementation sites were significantly more likely to have heard of it (83.9% vs. 70.5%). More respondents from implementation sites reported that they used THRIVE principles personally in their daily practice (58.5% vs. 49.0%). When completing a short test on their knowledge of the five domains of the THRIVE Framework, those in implementing sites were more likely to get perfect answers (34.1% vs. 22.9%).

| **Table S2** Staff Survey Results | | | | |
| --- | --- | --- | --- | --- |
| **Question** | **i-THRIVE** | **Comparison** | **p-value** | **Cramer’s V** |
| Awareness of THRIVE | 83.9% | 70.5% | <0.0001 | 0.15 |
| Personal use of THRIVE in daily practice | 58.5% | 49.0% | 0.03 | 0.12 |
| Perfect knowledge of THRIVE Framework | 34.1% | 22.9% | 0.001 | 0.12 |

Responses from each site were compared to the mean response from all comparison sites, for questions about site implementation of THRIVE, personal use of THRIVE principles, and exhibiting perfect knowledge of THRIVE principles (Tables S3—S5). Most i-THRIVE site respondents had a higher odds of reporting site implementation of THRIVE (all except Sites C and R), compared to the comparison site average (Table S3). Some comparison site respondents also had an increased odds of reporting site implementation of THRIVE (Sites J and K), compared to the comparison site average. When reporting personal use of THRIVE principles, there were no notable differences between respondents at any site and the comparison site average, except a lower odds of reporting personal use of THRIVE principles from respondents at Site F (Table S4). Many i-THRIVE site respondents had a higher odds of exhibiting perfect knowledge of THRIVE principles (Sites B, D, G, L, and Q), compared to the comparison site average. Site J (a comparison site) respondents also had an increased odds of exhibiting perfect knowledge of THRIVE principles, compared to the comparison site average (Table S5).

| **Table S3** Odds of Responders at Sites Reporting Site Implementation of THRIVE | | | |
| --- | --- | --- | --- |
| **Site Type** | **Sites** | **Odds Ratio** | **95% CI** |
| i-THRIVE | Site B | – | – |
|  | Site C | 0.59 | 0.10–3.37 |
|  | Site D | 4.71 | 1.76–12.57 |
|  | Site G | 8.83 | 1.91–40.77 |
|  | Site L | 4.51 | 1.69–12.09 |
|  | Site O | 4.91 | 1.84–13.05 |
|  | Site Q | 4.20 | 1.86–10.66 |
|  | Site R | 1.18 | 0.43–3.23 |
|  | Site T | 11.77 | 1.45–95.67 |
| Comparison | Site A | 0.51 | 0.19–1.40 |
|  | Site E | 0.82 | 0.24–2.82 |
|  | Site F | 0.35 | 0.07–1.85 |
|  | Site H | 3.07 | 0.74–12.71 |
|  | Site I | 0.31 | 0.08–1.23 |
|  | Site J | 4.43 | 2.32–8.47 |
|  | Site K | 4.43 | 1.33–14.80 |
|  | Site N | 0.55 | 0.17–1.77 |
|  | Site P | 1.78 | 0.78–4.04 |
|  | Site S | 0.35 | 0.04–3.51 |
| Referent | Mean of all comparison sites | 1.00 | – |
| * all respondents from Site B responded “yes”, thus an odds ratio could not be calculated  CI = confidence interval, | | | |

| **Table S4** Odds of Responders at Sites Reporting Personal Use of THRIVE Principles | | | |
| --- | --- | --- | --- |
| **Site Type** | **Site** | **Odds Ratio** | **95% CI** |
| i-THRIVE | Site B | – | – |
|  | Site C | 0.30 | 0.06–1.43 |
|  | Site D | 0.96 | 0.35–2.61 |
|  | Site G | 1.71 | 0.45–6.56 |
|  | Site L | 1.58 | 0.53–4.70 |
|  | Site O | 1.25 | 0.44–2.52 |
|  | Site Q | 0.92 | 0.37–2.40 |
|  | Site R | 0.87 | 0.27–2.77 |
|  | Site T | 1.58 | 0.31–8.00 |
| Comparison | Site A | 1.03 | 0.32–3.29 |
|  | Site E | 1.89 | 0.38–9.54 |
|  | Site F | 0.17 | 0.03–0.97 |
|  | Site H | 2.61 | 0.30–22.90 |
|  | Site I | 0.99 | 0.28–3.52 |
|  | Site J | 1.23 | 0.60–2.51 |
|  | Site K | 1.89 | 0.38–9.54 |
|  | Site N | 1.21 | 0.30–4.93 |
|  | Site P | 0.56 | 0.20–1.58 |
|  | Site S | 0.77 | 0.07–8.88 |
| Referent | Mean of all comparison sites | 1.00 | – |
| * all respondents from Site B responded “yes”, thus an odds ratio could not be calculated  CI = confidence interval | | | |

| **Table S5** Odds of Responders at Sites Having Perfect Knowledge of THRIVE  Perfect knowledge was defined as achieving a perfect score on the THRIVE quiz. | | | |
| --- | --- | --- | --- |
| **Site Type** | **Site** | **Odds Ratio** | **95% CI** |
| i-THRIVE | Site B | 3.60 | 1.21–10.70 |
|  | Site C | 1.54 | 0.31–7.71 |
|  | Site D | 2.70 | 1.27–5.72 |
|  | Site G | 2.70 | 1.02–7.13 |
|  | Site L | 5.06 | 2.30–11.10 |
|  | Site O | 1.90 | 0.90–4.01 |
|  | Site Q | 11.24 | 5.19–24.31 |
|  | Site R | 0.75 | 0.30–1.89 |
|  | Site T | 1.47 | 0.39–5.53 |
| Comparison | Site A | 0.45 | 0.15–1.35 |
|  | Site E | 0.34 | 0.10–1.15 |
|  | Site F | 0.69 | 0.15–3.16 |
|  | Site H | 0.65 | 0.14–2.92 |
|  | Site I | 1.39 | 0.59–3.30 |
|  | Site J | 3.73 | 2.25–6.19 |
|  | Site K | 1.15 | 0.37–3.60 |
|  | Site N | 1.92 | 0.71–5.20 |
|  | Site P | 1.57 | 0.71–3.48 |
|  | Site S | 0.81 | 0.18–3.75 |
| Referent | Mean of all comparison sites | 1.00 | – |
| CI = confidence interval | | | |

**THRIVE Fidelity**

For fidelity scores, the interrater reliability (Krippendorf’s alpha) among the raters ranged from 0.57 to 0.93, with a mean alpha of 0.73.

Unweighted group characteristics are in Table S6, with the weighted standardised difference in means. An important indicator of balanced group characteristics is a standardised difference in means of less than 0.25 for all characteristics and groups, with the pre-implementation i-THRIVE group serving as the baseline for comparison.

| **Table S6** Four-Group Propensity Score Weighted Characteristics of i-THRIVE and Comparison Sites | | | | | | | | |
| --- | --- | --- | --- | --- | --- | --- | --- | --- |
| **Characteristics** | **Mean (SD)** | | | | | **Weighted standardised difference in means, compared to Pre i-THRIVE** | | |
|  | **i-THRIVE** | | **Control** | | | **Post i-THRIVE** | **Pre control** | **Post control** |
|  | **Pre**  (n=10) | **Post**  (n=10) | | **Pre**  (n=10) | **Post**  (n=10) |  |  |  |
| Population density (persons per 10 km^2^) | 49.7  (47.2) | 52.2  (51.5) | | 23.9  (29.0) | 24.5  (29.8) | 0.061 | 0.253 | 0.165 |
| Funding  (£100,000) | 47.3  (47.4) | 60.4  (41.4) | | 44.3  (21.1) | 56.2  (34.9) | 0.018 | 0.210 | 0.067 |
| IMD | 84.0  (60.4) | 93.8  (51.8) | | 69.1  (44.7) | 67.8  (45.5) | 0.056 | 0.292 | 0.256 |
| Baseline Number of CCGs | 1.4  (1.0) | 1.4  (1.0) | | 1.7  (1.3) | 1.7  (1.3) | 0.039 | 0.000 | 0.200 |
| Transformation progress | 72.0  (27.0) | 72.0  (27.0) | | 64.0  (20.0) | 64.0  (20.0) | 0.108 | 0.265 | 0.383 |
| CCG = Clinical Commissioning Group, IMD = Indices of Multiple Deprivation, Post = post-implementation, Pre = pre-implementation, SD = standard deviation | | | | | | | | |

## Supplemental Material S8: i-THRIVE Effect Moderation

This section of the supplemental materials details the approach used to investigate the influence of effective working relationships among local systems on the impact of the i-THRIVE programme in enhancing fidelity.

**Working Relationships as a Moderating Factor**

The i-THRIVE model emphasizes the importance of strong working relationships at various levels. At the macro level, it encourages cooperation among various agencies, such as educational and social services, in policy making and care commissioning (Moore *et al.* 2023). At the meso level, a THRIVE-aligned site would typically feature a network of community providers (Moore *et al.* 2023). Achieving these objectives is significantly influenced by the effectiveness of working relationships at the site as well as within the broader community, including supporting agencies. These collaborations are essential for the successful implementation of i-THRIVE and are largely contingent upon the willingness and interest of these agencies to participate in CAMHS activities.

Given this context, we explored the role of effectiveness of working relationships among local systems. Our hypothesis was that effectiveness of working relationships would likely affect macro- and meso-level fidelity scores and potentially the overall fidelity score, though not on micro-level fidelity scores.

**Data and Methods**

We utilized the four-group propensity-score weights derived from the sensitivity analyses (see Supplementary Materials S6). To gauge effectiveness of local system working relationships at each site, we used the "effectiveness of local system working relationships" component rating from the CCG Assurance Annual Assessment 2017/18 (NHS England 2021). We averaged these ratings for sites with multiple CCGs. A threshold of 68.0 was set to distinguish sites with highly effective local system working relationships. The mean overall score was 68.9 for comparison sites and 68.3 for implementation sites, with five highly effective comparison sites (Sites E, I, N, P, and S) and five highly effective implementation sites (Sites D, L, M, O, and T) identified.

We employed a difference-in-difference-in-differences (DiDiD) model to assess i-THRIVE's impact on macro-level fidelity. This analysis was conducted using maximum-likelihood repeated measures linear regression with an auto-regressive correlation structure, weighted with propensity scores. The model included a three-way interaction of variables representing highly effective working relationships, the post-implementation period, and the intervention group. To account for any remaining differences in characteristics, we included the covariates population density, IMD rank, and transformation compliance in the final model. This methodological approach allows us to discern the moderating effect of working relationship effectiveness on the impact of i-THRIVE, providing a more comprehensive understanding of the factors contributing to successful implementation.

## References

**Auguie B** (2017) *gridExtra: Miscellaneous functions for ‘Grid’ graphics* (manual). Retrieved from https://CRAN.R-project.org/package=gridExtra

**Chang W** (2023) *extrafont: Tools for Using Fonts* (manual). Retrieved from https://CRAN.R-project.org/package=extrafont

**Department for Communities and Local Government** (2015) English Indices of Deprivation 2015 File 5 Scores for the Indices of Deprivation.

**Department for Communities and Local Government** (2019) English Indices of Deprivation 2019 File 5 Scores for the Indices of Deprivation.

**Dunnington D** (2023) *ggspatial: Spatial data framework for ggplot2* (manual). Retrieved from https://CRAN.R-project.org/package=ggspatial

**FC M, Davis TL and ggplot2 authors** (2022) *ggpattern: ’ggplot2 Pattern Geoms* (manual). Retrieved from https://CRAN.R-project.org/package=ggpattern

**Gamer M, Lemon J and ¡puspendra.pusp22@gmail.com¿ IFPS** (2019) *irr: Various coefficients of interrater reliability and agreement* (manual). Retrieved from https://CRAN.R-project.org/package=irr

**Hayes AF and Krippendorff K** (2007) Answering the Call for a Standard Reliability Measure for Coding Data. *Communication Methods and Measures* **1**(1), 77–89. https://doi.org/10.1080/19312450709336664.

**i-THRIVE Team** (n.d.) *i-THRIVE Accelerator Sites*. Retrieved from https://implementingthrive.org/implementation-sites/i-thrive-accelerator-sites/

**Moore A, Lindley Baron-Cohen K, Simes E, Chen S and Fonagy P** (2023) A protocol for a multi-site cohort study to evaluate child and adolescent mental health service transformation in England using the i-THRIVE model. *PLoS ONE* **18**(5), e0265782. https://doi.org/10.1371/journal.pone.0265782.

**National i-THRIVE Programme Team** (2017) *i-THRIVE: Year One of Implementation Annual Report May 2017* (Annual). Retrieved from https://www.implementingthrive.org/wp-content/uploads/2017/05/i-THRIVE-Annual-Report-2017.pdf

**Neuwirth E** (2014) RColorBrewer: ColorBrewer Palettes. (Version R package version 1.1-2).

**NHS England** (2017, January 9) Mental Health Five Year Forward View Dashboard. (Version 1.1). Online. https://view.officeapps.live.com/op/view.aspx?src=https%3A%2F%2Fwww.england.nhs.uk%2Fwp-content%2Fuploads%2F2017%2F01%2Fmhfyfv-dashboard-q4-1617.xlsx&wdOrigin=BROWSELINK (accessed 1 June 2023)

**NHS England** (2019a, January 5) CCG Allocations 2019/20 to 2023/24 - Technical Guidance. NHS England. https://view.officeapps.live.com/op/view.aspx?src=https%3A%2F%2Fwww.england.nhs.uk%2Fwp-content%2Fuploads%2F2019%2F05%2Fxchanges-to-ccg-dco-stp-mappings-over-time.xlsx&wdOrigin=BROWSELINK

**NHS England** (2019b, January 5) Changes to CCG-DCO-STP Mappings Over Time. https://view.officeapps.live.com/op/view.aspx?src=https%3A%2F%2Fwww.england.nhs.uk%2Fwp-content%2Fuploads%2F2019%2F05%2Fxchanges-to-ccg-dco-stp-mappings-over-time.xlsx&wdOrigin=BROWSELINK

**NHS England** (2021, April 2) CCG Improvement and Assessment Framework (CCG IAF) Data Extract 2017/18. https://view.officeapps.live.com/op/view.aspx?src=https%3A%2F%2Fwww.england.nhs.uk%2Fwp-content%2Fuploads%2F2017%2F07%2Fccg-iaf-data-extract-crosstab-20201118.xlsx&wdOrigin=BROWSELINK

**Noble S, McLennan D, Noble M, Plunkett E, Gutacker N, Silk M and Wright G** (2019, September) The English Indices of Deprivation 2019. Ministry of Housing, Communites and Local Government.

**Office for National Statistics** (2016, September 28) CCG (July 2015) Ultra Generalised Clipped Boundaries in England. ONS Geography. https://geoportal.statistics.gov.uk/datasets/ons::ccg-july-2015-ultra-generalised-clipped-boundaries-in-england/explore?location=52.821499%2C-2.455538%2C7.12

**Office for National Statistics** (2017, October 9) CCG (April 2018) Ultra Generalised Clipped Boundaries in England. ONS Geography. https://geoportal.statistics.gov.uk/datasets/ons::ccg-apr-2018-ultra-generalised-clipped-boundaries-in-england/explore

**Pebesma E** (2018) Simple features for R: Standardized support for spatial vector data. *The R Journal* **10**(1), 439–446. https://doi.org/10.32614/RJ-2018-009.

**Pebesma E and Bivand R** (2023) *Spatial data science: With applications in R*. Chapman and Hall/CRC. https://doi.org/10.1201/9780429459016.

**Stuart EA, Huskamp HA, Duckworth K, Simmons J, Song Z, Chernew ME and Barry CL** (2014) Using propensity scores in difference-in-differences models to estimate the effects of a policy change. *Health Services and Outcomes Research Methodology* **14**(4), 166–182. https://doi.org/10.1007/s10742-014-0123-z.

**Venables WN and Ripley BD** (2002) *Modern Applied Statistics with S*, 4th Edition. New York: Springer.

**Wickham H** (2007) Reshaping Data with the reshape Package. *Journal of Statistical Software* **21**(12), 1–20.

**Wickham H, Averick M, Bryan J, Chang W, McGowan L, François R, Grolemund G, Hayes A, Henry L, Hester J, Kuhn M, Pedersen T, Miller E, Bache S, Müller K, Ooms J, Robinson D, Seidel D, Spinu V, Takahashi K, Vaughan D, Wilke C, Woo K and Yutani H** (2019) Welcome to the Tidyverse. *Journal of Open Source Software* **4**(43), 1686. https://doi.org/10.21105/joss.01686.

**Wickham H and Bryan J** (2023) readxl: Read Excel Files. (Version 1.4.2) [R]. https://CRAN.R-project.org/package=readxl

**Wickham H and Miller E** (2017) haven: Import and Export ‘SPSS’, ‘Stata’ and ‘SAS’ Files. (Version 1.1.0) [R].

**Wilke CO** (2020) cowplot: Streamlined plot theme and plot annotations for ‘ggplot2’. [R]. https://CRAN.R-project.org/package=cowplot

**Yoshida K and Bartel A** (2022) *tableone: Create ‘table 1’ to describe baseline characteristics with or without propensity score weights* (manual). Retrieved from https://CRAN.R-project.org/package=tableone
